# “When people who use drugs can’t differentiate between medical care and cops, it’s a problem.” Compounding risks of law Enforcement Harassment & Punitive Healthcare Policies

**DOI:** 10.1186/s40352-023-00256-3

**Published:** 2024-02-06

**Authors:** Bayla Ostrach, Vanessa Hixon, Ainsley Bryce

**Affiliations:** 1 Boston University School of Medicine; Fruit of Labor Action Research & Technical Assistance, LLC, Fairview, NC USA; 2 Appalachian Medical Solidarity, Asheville, North Carolina, USA; 3 Holler Harm Reduction, Marshall, NC USA

## Abstract

**Background:**

Community-based harm reduction programming is widely recognized as an effective strategy for reducing the increased risks for and spread of HIV, HCV, and for reducing the growing rate of overdose deaths among people who use drugs (PWUD). PWUD in the United States (US) are a highly justice-involved population, also at increased risk for law enforcement interaction, arrest, and incarceration. These risks compound and interact in the context of criminalization and law enforcement surveillance. Justice involvement increases risks for overdose and for riskier injecting behavior among PWUD, in turn increasing HCV and HIV risks. In Central and Southern Appalachia specifically, PWUD have identified fear of law enforcement harassment and arrest as a barrier to engaging in harm reduction behavior, and a deterrent to seeking help at the scene of an overdose. Moreover, stigmatizing and punitive treatment in healthcare settings can deter PWUD from seeking care, with life or death consequences. This evaluation research study assessing the successes and impacts of a grant-funded project to increase access to safer drug consumption supplies and overdose prevention education for PWUD, including justice-involved participants of a syringe access program (SAP), in public housing and beyond in a South-Central Appalachian setting used key informant and opportunistic sampling. Mixed-methods data were compiled and collected including secondary program data; primary interview and participant-observation data.

**Results:**

The evaluation research identified that grant deliverables were largely achieved, despite challenges presented by the COVID-19 pandemic. In addition, SAP participants and staff reported larger themes surrounding grant-funded activities, in which they perceived that widespread local law enforcement harassment of PWUD increased participants’ risks for overdose death and infectious disease risks and that punitive local healthcare settings and policies acted as deterrents to care-seeking for many PWUD.

**Conclusions:**

Overall, the evaluation research found that participants’ experiences with and perceptions of local law enforcement harassment combined with their understandings and experiences of local punitive healthcare settings and policies; together compounding and increasing overdose risks and negative health consequences for local justice-involved PWUD.

People who use drugs (PWUD) in the United States experience increased susceptibility to and severity of COVID-19, as well as increased incidence of Hepatitis C (HCV) (Kattakuzhy & Rosenthal, 2020). HIV diagnoses are increasing nationally among people who inject drugs, specifically, with the COVID-19 pandemic likely exacerbating risks for HIV transmission such as lack of access to adequate safer drug consumption supplies, recent incarceration and/or being unsheltered, and/or co-infection with HCV (CDC, 2020). Overdose deaths among PWUD also reached historically unprecedented levels, exceeding 100,000 in a 12-month period by early 2021 (CDC, 2021). Recent incarceration is one of the greatest risk factors for overdose (Binswanger & Glanz, 2018; Binswanger, Nguyen, Morenoff, Xu, & Harding, 2020; Ranapurwala et al., 2018).

People who use drugs in the U.S. are also, by definition, a highly justice-involved population, at increased risk for law enforcement interaction, arrest, and incarceration. Given the long-term criminalization of non-prescribed drug use in most of the United States, simply engaging in the use of substances places PWUD at risk for law enforcement surveillance, intervention, and criminal justice involvement (Singer, 2007; Singer & Page, 2016). As of 2018, nearly 1 in 7 state prisoners were people whose most serious charge was drug-related (Caulkins & Reuter, 2021). By 2015, almost a third of U.S. adults ages 24–34 reported being arrested at least once in their lifetime, with rates disproportionately higher for Black and Indigenous people, and almost twice as high for men as for women (Barnes, Jorgensen, Beaver, Boutwell, & Wright, 2015). Though specific statistics are not readily available to estimate rates, simply by virtue of the criminalization of substance use, PWUD in the U.S. are heavily stigmatized, surveilled, and scapegoated (Singer, 2007; Singer & Page, 2016) and thus may be arrested and incarcerated at higher rates as compared to the overall population (Brinkley-Rubinstein, Cloud, Drucker, & Zaller, 2018a).

Justice involvement, particularly in the form of law enforcement surveillance and encounters, measurably increases risks for overdose and for riskier injecting behavior, in turn increasing HCV and HIV risks (Baker et al., 2020; Banta-Green, Beletsky, Schoeppe, Coffin, & Kuszler, 2013; Bohnert et al., 2011; Wagner, Simon-Freeman, & Bluthenthal, 2013)**.** The effects of policing on health risks for PWUD are so strong that law enforcement has been referred to as a structural determinant of health for syringe service program clients (Beletsky et al., 2015). Multiple arrests, in particular, increase overdose death risk (Ahlner, Holmgren, & Jones, 2016)**.** Carceral health scholars describe a “criminal justice continuum” affecting PWUD, specifically opioid users, and their related risk for overdose at various points along it (Brinkley-Rubinstein et al., 2018b). In Central and Southern Appalachia specifically, PWID identified fear of law enforcement harassment and arrests as one of the greatest barriers to accessing sterile drug consumption supplies (Carpenter et al., 2022; Davis et al., 2019).

In some settings, fear or threat of law enforcement surveillance and arrest may act as a deterrent to seeking assistance at the scene of an overdose. This appears to be particularly true where local or state policy or law can be used to hold a person present at the scene of an overdose responsible for a fatality, through so-called “drug-induced homicide” charges (Carroll, Green, & Noonan, 2018; Hamilton et al., 2022; Rouhani et al., 2021). Experience with such laws, even hearing about them second-hand, appears to have the effect of potentially undermining Good Samaritan 911 laws intended to encourage those present at an overdose to seek medical aid (J. J. Carroll et al., 2021).

Negative treatment in other settings can also deter PWUD from seeking medical aid, compounding overdose risks. Stigma and biased treatment by medical professionals and healthcare providers are recognized as a major public health issue (Paquette, Syvertsen, & Pollini, 2018) that compounds and contributes to disease interactions, increasing health risks for substance users and their wider communities (Singer & Ziegler, 2017). Stigma enacted toward substance users by healthcare providers and in medical settings has life or death consequences, as stigmatized PWUD are less likely to access and/or complete care (Paquette et al., 2018). When PWUD leave healthcare settings ‘against medical advice’ (AMA) they often do so due to reportedly poor treatment by hospital staff rather than because they were unwilling to receive care (Jafari et al., 2015)**.** Healthcare settings with punitive or discriminatory policies and treatment produce risk environments that deter PWUD from seeking care (McNeil, Small, Wood, & Kerr, 2014; Meyerson et al., 2021).

Yet well-established, evidence-based, proven approaches to improve health and reduce risks for PWUD and the wider communities in which they live, exist. Community-based harm reduction, especially when provided through syringe service programs that offer a wide variety of safer drug consumption supplies, naloxone, mobile distribution of supplies; and that encourage PWUD to report back peer reversals of overdoses, is widely recognized as an effective strategy for reducing the spread of HIV, HCV, and for reducing overdose deaths (Bryce et al., 2021; Carroll et al., 2018; Des Jarlais et al., 2005; Linas et al., 2021; Naumann et al., 2019; Small, Glickman, Rigter, & Walter, 2010; Strike & Miskovic, 2018; Wodak et al., 2004).

## Objectives

Using mixed-methods data collected during an evaluation study originally designed to assess the completion of grant-funded activities at a Southern Appalachia community-based syringe access program serving a highly marginalized population of PWUD the authors detected broader research questions informed by the evaluation findings. Grounded in those evaluation data, the objective of this article is thus to illuminate program participants’ intertwined experiences with and perceptions of law enforcement interactions and healthcare settings.

## Methods

### Funded project

From September 2019 through March 2021 a community-based harm reduction organization operating a syringe service program in Southern Appalachia (hereafter referred to as “Blue Ridge SAP”) received two parallel grants from the same funder to support a) ongoing distribution of safer drug consumption supplies and scale up mobile distribution of these supplies in public housing areas and b) to educate community members and faith leaders about how to use naloxone to reverse overdoses and save lives and the importance of advocating for the legal and human rights of justice-involved PWUD in local policy, policing, and healthcare climates. An external evaluation of the funded project was conducted prior to its conclusion, forming the basis of the data for this paper.

### Setting

Evaluation data collection took place in a mostly urban setting in Southern Appalachia (USDA Economic Research Service, 2020), in collaboration with Blue Ridge SAP (staffed at the time by two of the authors) but led by a contracted outside evaluator (the first author). Blue Ridge SAP distributes safer drug consumption and overdose prevention and reversal supplies in mostly urban areas of one large county in Southern Appalachia (USDA Economic Research Service, 2020). For this urban/rural designation, we use the 7-category rural-urban commuting area (RUCA) code (USDA Economic Research Service, 2020). RUCA codes, based on census tract, encompass population density, commuting patterns, and adjacency to other densely populated areas. While not a perfect measure for this study given the marginalized status of the largely unhoused or precariously housed and largely justice-involved participants served by Blue Ridge SAP (for whom commuting is a less relevant factor, for example), in this case the RUCA designation is somewhat more descriptive than other options, such as the Index of Relative Rurality (Kaiser Family Foundation, 2017) which classifies the area in question as “other” (in between rural and urban).

### Human subjects review

A study protocol for open-ended, mixed-methods data collection about harm reduction efforts and structural, policy, and locally specific risk factors for overdose and infectious disease including the role of healthcare and law enforcement with harm reduction/syringe access program staff and participants was previously reviewed and found exempt from further review by the Institutional Review Board at a local hospital. All Blue Ridge SAP participants were made aware of primary data collection periods, including periods of participant-observation and informed of their right to participate or not; participants were also assured they could receive supplies whether or not they participated in data collection. Participants in informal interviews gave explicit verbal consent for (de-identified) accounts they shared to be used for evaluation purposes, as well as disseminated more broadly.

### Sampling & recruitment

Evaluation data consisted of a combination of primary and secondary data (Tables 1 and 2) gathered through a mix of purposive, key informant, site-based, and opportunistic sampling. Blue Ridge SAP shared de-identified secondary program data that included participant demographics and supply distribution tracking overall and by outreach site from throughout the funded period. We were also granted access to recruit program participants and staff for interviews and to attend outreach locations to engage in participant-observation for primary data collection toward the end of the funded period.

**Table 1:** Data sources

Data sources	
Primary Data (2/2021–3/2021)	Secondary Data (9/2019–3/2021)
Nested samples, n ~ 260 program participants & *n* = 3 program staff **•** Informal interviews with syringe access program participants, *n* = 10 **•** Semi-structured interviews and focus groups with syringe access program staff, *n* = 3 **•** Participant-observation at three syringe access program outreach sites, *n* = 250+ regular participants	Nested sample from funded grant period, *n* = 1905 **•** De-identified participant demographic data from funded period **•** Safer drug consumption supply distribution data **•** Participant-reported overdose reversal data

**Table 2:** Participant characteristics for secondary (program) data

Data source	Total unique participants	White	Black	Latinx	AI/NA	Asian	% BIPOC
Secondary program data							
** Public Housing 1 outreach site**	185	138	38	9	0	0	25%
Public Housing 2 outreach site*	8	7	1	0	0	0	13%
Public Housing 2 outreach site*	12	11	1	0	0	0	8%
Public housing, combined							26%
**Unhoused shelter outreach site**	67	52	14	1	0	1	22%
**Downtown outreach site**	824	732	76	13	3	0	11%
**Progressive local business outreach site**	605	559	35	10	1	0	8%
unspecified site						1	n/a
**Unduplicated participants**	1905	*(program participants otherwise may repeat across outreach sites)*					

* ceased outreach during COVID-19; i.e.*, for most of funded period*

### Data collection

The first author conducted all data collection; other authors participated in data analysis and interpretation. We recruited study participants for primary data collection from among Blue Ridge SAP participants and staff, following a notification period in which all outreach sites attendees were made aware that evaluation activities would be upcoming. Originating in a grant evaluation, initially we asked Blue Ridge SAP participants and staff about topics specific to the scope of the grant: safer drug consumption supply distribution; mobile distribution; impacts of expanded supply distribution in public housing areas; and efforts to educate community members and faith leaders about overdose recognition and reversal and advocacy for the legal and human rights of justice-involved PWUD. Similarly, secondary program data were initially assessed for progress toward grant objectives and fulfillment of funded activities.

During on-site data collection periods, the first author approached SAP participants to confirm they were aware of the evaluation and confirmed verbal consent to discuss evaluation topics and for information they shared to be used and shared, de-identified. In all cases the first author approached participants only after they had obtained their supplies that day, to avoid any appearance of coercion or quid pro quo. Data collection with participants largely took the form of informal interviews on-site at outreach locations. The first author also conducted semi-structured interviews with SAP staff.

### Data analysis

Data were analyzed using a combination of descriptive statistics (secondary data) to generate a demographic profile of Blue Ridge SAP participants during the study period, in particular at different outreach sites as compared to overall, and thematic analysis of primary data. The first author, an experienced qualitative researcher, hand-coded and analyzed memos from all informal interviews; detailed notes from participant-observation; and relevant policy documents using a codebook based on a combination of existing relevant literature; grant deliverables; suggestions from SAP staff about salient, timely factors; and familiarity with the local context based on participant-observation. The codebook was member-checked with SAP staff. Coded materials were then closely read multiple times to detect emerging themes and relationships between codes; and then to theorize relationships between the themes (Creswell, 2013). Following traditions of modified Grounded Theory (Charmaz, 2006), the authors followed the direction in which participants steered the data during both data collection and analysis. As the authors analyzed and began to interpret, synthesize, and then triangulate the evaluation data, initial evaluation findings informed larger topics of interest beyond Blue Ridge SAP’s immediate grant reporting and program planning.

## Results

### Blue ridge SAP outreach sites

During the funded period; and thus, the period for which secondary program data were available for analysis, Blue Ridge SAP operated at a total of six sites, though not at all of them for the entire period (as the funded period largely coincided with the beginning of the COVID-19 pandemic, distribution at non-mobile sites ceased). The continuous sites throughout the period for which data were available were at or outside of a downtown location co-located with other community services available to unhoused and marginalized county residents (hot meal; first aid/wound care; some linkage to care); at or outside of a local progressive business; and at a public housing area location newly established under the grant.

Other outreach sites during some portion of the period for which data were available (typically the six pre-COVID months) were two other public housing sites with much lower populations; and onsite at a day shelter for unhoused county residents that also has an onsite primary care clinic.

### Participant characteristics

During the funded period, based on the most complete secondary program data available from supply distribution encounters across all outreach sites, there were an overall total of about 1900 unduplicated Blue Ridge SAP participants. Of these, approximately 13% were BIPOC; in a county where the overall BIPOC population was approximately 17% as of 2021 (based on Census Data; direct source suppressed to preserve participant confidentiality). However, the proportion of BIPOC participants varied at different outreach sites. In the county where Blue Ridge SAP is based and distributes safer drug consumption supplies, an estimated 85% of residents in public housing are Black (*suppressed for confidentiality*), as compared to an overall Black population of about 6% in the surrounding county (*suppressed for confidentiality*). During the grant evaluation period the proportion of BIPOC participants at Blue Ridge SAP public housing outreach sites represented an average of about 15%; ranging from 13% at the least active site to 25% at the new site funded under the grant being evaluated. At other locations the proportion of BIPOC participants served during the period for which data were made available varied from 22% (at the unhoused shelter day services site); to 11% (at the downtown location); to 8% (at the progressive local business). During the first year of the funded period, program staff calculated that the addition of the new outreach site in the larger public housing site increased the proportion of BIPOC participants served by 85%; however the specific baseline number or percentage of BIPOC participants prior to the period for which data were made available for this analysis was not made available to these authors.

While specific data on arrests or recent incarceration are not systematically collected at the time of supply distribution SAP staff reported that the majority of Blue Ridge SAP program participants had a history of justice-involvement. In particular, what this most often consisted of was having been arrested for possession of drug paraphernalia as local law enforcement routinely disregarded a limited immunity clause in the NC syringe exchange law (G.S.90-113.22. Possession of Drug Paraphernalia, 2019) that provides for immunity from arrest for possession of injecting supplies, including used syringes with small amounts of residue, when the person stopped by law enforcement presents a card indicating they are a participant of an SSP registered with the state (as Blue Ridge SAP is).During participant-observation for the grant evaluation the first author observed that during all outreach shifts multiple Blue Ridge SAP participants reported recent encounters with local law enforcement in which they presented a valid SSP participant card and were arrested for paraphernalia anyway, and even incidents in which local law enforcement tore up their participant cards. Moreover, all SAP participants that participated in informal interviews for the grant evaluation had been arrested; several described their arrests and experiences in jail in detail. SAP staff from whom data were collected included one woman and two nonbinary individuals, all white; at least one had a history of arrest.

### Program activities

During the grant evaluation period Blue Ridge SAP expanded distribution of safer drug consumption and overdose prevention supplies in multiple public housing sites in an area of South-Central Appalachia that had experienced disproportionately high overdose rates and intensive policing and surveillance of PWUD. During the same time period Blue Ridge SAP also largely maintained existing supply distribution sites while adapting for the unexpected COVID-19 pandemic environment and constraints. Despite the pandemic, participation (as measured by unique participants) was up by an estimated 55%. With efforts to ensure adequate syringe coverage to compensate for quarantine and isolation periods in mind, syringe distribution increased during grant period as compared to preceding time periods, by an estimated 90%. The number of participant-reported overdose reversals using kits provided by Blue Ridge SAP during the grant period as compared to prior years was not significantly lower despite known pandemic impacts on highly justice-involved PWUD being more likely to use alone (Ostrach et al., 2021).

During the grant period, in an unprecedented expansion of services, Blue Ridge SAP began distributing safer smoking supplies; primarily in public housing areas. Blue Ridge SAP’s expansion in public housing and maintenance of existing sites, made possible by offering mobile distribution during COVID-19, as well as the addition of supplies for more routes of drug ingestion together resulted in notable demographic changes in the Blue Ridge SAP participant population with increases in supply distribution Black and Latinx participants, with a smaller increase in Asian participants. Supply distribution to unique Black participants increased by more than 80% from the same period of time prior to receipt of grant funding. These changes were largely attributed to the SAP’s increased presence in public housing.

When asked broad questions about supply distribution many Blue Ridge SAP program participants, especially those receiving supplies at outreach sites located in public housing, spoke about wider structural, policy, and local contextual issues. Beyond the grant evaluation findings, broader themes thus emerged from participant experiences and perspectives. Key themes were that *law enforcement harassment increases overdose death and infectious disease risks; punitive healthcare settings and policies act as deterrents to care-seeking;* which informed the larger overarching theme that *law enforcement harassment & punitive healthcare settings and policies intersect to compound negative experiences and risks.*

### Law enforcement harassment increases overdose death risks

Blue Ridge SAP program participants specifically described how local law enforcement routinely violate the limited immunity clause of NC’s Good Samaritan overdose reversal and syringe exchange laws by arresting PWUD at the scene of an overdose when they seek assistance and arresting people for paraphernalia even when they show a valid participant card for a registered SAP. Participants described to us during the grant evaluation, and routinely reported to SAP staff and volunteers, that such experiences widely lead them to avoid calling 911 in situations of overdose and medical emergencies and cause widespread fear of arrest. Participants were widely aware of NC’s Good Samaritan and the limited immunity clause in the syringe exchange law but were also quick to mention that local law enforcement often do not respect these laws, which also appeared to lead to participants not differentiating between legality of syringes and the comparative illegality of safer smoking supplies (which are not currently covered by the limited immunity statute). This confusion also contributed to participants not necessarily knowing what activities potentially higher risk were for being charged, following an arrest. Intensifying participants’ fears of being arrested and charged following their own overdose or when helping to reverse someone’s overdose, despite the Good Samaritan law, a then-recently passed ‘Death by Distribution’ law compounded the perception that seeking emergency services for an overdose would result in law enforcement response rather than assistance. Participants already fearful of local law enforcement not respecting the limited immunity clauses of the state’s Good Samaritan naloxone law and syringe exchange law also began to fear that in addition to being arrested for paraphernalia for unused intramuscular naloxone syringes in overdose reversal kits or when calling 911 from the scene of an overdose, law enforcement also might accuse them of murder or homicide for being present at a fatal overdose. Blue Ridge SAP participants described that this was already beginning to happen to other PWUD they knew, as had been documented in other parts of their region (J. J. Carroll et al., 2021). At the time these data were collected, the NC Good Samaritan naloxone law excluded fentanyl from limited immunity protections, meaning someone was not protected from arrest when calling 911 to seek help in the event of a fentanyl overdose (Dixon, 2023). These concerns were reported in informal interviews conducted for the grant evaluation; were frequently mentioned during outreach shifts while participant-observation occurred; and were also confirmed by Blue Ridge SAP staff interviewed.

For example, during the grant evaluation period three precariously housed participants attending the SAP participated in a group interview in which they described their participant cards being torn up by law enforcement in front of them. One described law enforcement officers ignoring and tearing up his participant card while arresting him for paraphernalia – at the scene of an overdose. The participant describing the incident told us he called 911 when a person in his car overdosed and that he was arrested even after he declared the syringes in his car and mentioned his participant card, as required:

*[the police] arrived and yanked me out of the car, [they] cuffed me, slammed me onto the hood of the car before [administering aid to the OD victim], they searched my car and found rigs… [me and my girlfriend] told the officer where to find our [SAP] participant cards in the vehicle but [the cop] ignored the cards.*

The participant reported a law enforcement officer threatened to take his car and told him, *“rigs aren’t just rigs in [his] car,”* and that *“[the] syringe exchange law doesn’t apply to [him] in the same way,”* after which he was arrested on paraphernalia charges – that were subsequently dropped when he went to court and showed his participant card. However, he reported the participant and the two other people with him at the time now avoid calling 911. They also said law enforcement harassment makes it harder to pick up overdose and infection prevention supplies from the SAP; harder to call 911 in the event of an overdose; and, because they are stopped by law enforcement repeatedly even sometimes more than once while walking down the same street, “*it’s harder to go where [we] need to*.”

The participant who had been arrested at the scene of the overdose stated, *“I would be more likely to call 911 [for an overdose] if I hadn’t been cuffed, searched, and charged after doing so.”* He said he would be more likely to call 911 in the future if limited immunity laws were followed: *““Cops make their own rules… if you disclose having rigs they use it as an excuse to search your car and find something to charge you for.“* In describing concerns about how law enforcement harassment contributed to overdose death risks, participants that were informally interviewed or sharing their perspectives during participant-observation frequently said, “*when I call 911, police come first* [before EMS].” A staff person from Blue Ridge SAP estimated local law enforcement ignored participant cards (that should confer limited immunity from paraphernalia arrests), “*about 40% of the time.*” This staff person said that when she offers participant cards to participants many say, “*I’ll take it but it doesn’t mean anything [to law enforcement*].”

The same staff person described a situation when a participant was overdosing and the people with the person overdosing called her, instead of 911. The staff person’s understanding at the time was the participants were scared to call 911 and have law enforcement arrive. She reported that everyone present had warrants and “*knew law enforcement wouldn’t respect the Good Samaritan law*.” Another SAP staff person stated, “*the Death by Distribution [law] is a total deterrent to calling 911 for overdose*.”

In addition, unsheltered Blue Ridge SAP program participants informally interviewed for the grant evaluation reported being repeatedly ticketed for panhandling, often multiple times in the same day, and at times adding up to enough tickets to result in arrest warrants and jail time. They described having no way to pay panhandling tickets and thus spending days in jail - which they were aware in turn increased their overdose risks. Two participants in particular perceived and described such arrests that would inevitably result in a few days in jail as deliberate attempts by local law enforcement to kill unsheltered PWUD by increasing their chances for a fatal overdose upon release.

Across all of these descriptions, participants and SAP staff that worked with them described law enforcement harassment of PWUD as a factor that increases overdose death risks, by reducing participant access to and comfort using emergency services.

### Punitive healthcare settings and policies deter care-seeking

In addition to law enforcement harassment that increased overdose death risks for justice-involved PWUD, participants providing data for the grant evaluation described local punitive healthcare policies and settings that deterred them from seeking medical care and substance use disorders treatment. In such descriptions, participants nearly conflated punitive healthcare settings and carceral settings, based on the punitive treatment they received in both spheres.

Blue Ridge SAP participants and staff frequently mentioned a well-known local policy established at and enforced by the local safety net hospital, known as the “Drug Abuse Protocol,” under which patients suspected of injection drug use or with a record of substance use disorder, past substance use, or substance use disorder treatment (including prescribed medication for opioid use disorder noted in their chart currently or in the past) are automatically placed on the equivalent of a psychiatric hold: denied visitors, denied access to their cell phone; disallowed outside food, use of metal utensils or straws; disallowed street clothing or any underwear and instead allowed only paper scrubs; and kept in a locked unit of the hospital. At the time of the grant evaluation this protocol was widely known among local service and healthcare providers; some harm reduction-oriented providers with privileges at the hospital had attempted to resist it or have patients they saw in the hospital exempted from it; while hospital employees concerned about it had also leaked copies of the protocol itself to employees of multiple local harm reduction organizations and to the media (*suppressed for confidentiality*). When asked about the draconian nature of the protocol hospital leadership defended it as ‘necessary for employee safety.’

One SAP staff person, a former hospital employee very familiar with the protocol, reported hospital staff were trained, “*never to turn their back*” on a patient placed under the protocol. She described a Blue Ridge SAP participant that had been admitted inpatient for three months, who, once placed on the protocol, was not allowed to see his young children – this was prior to COVID-19. Participants informally interviewed for the grant evaluation, and attending outreach sites during participant-observation, frequently mentioned not wanting to go to the hospital even in the event of overdose or severe infection or illness, due to the protocol: “*I don’t want all my stuff taken away*.” As a result, both participants and SAP staff reported people tend to be much sicker by the time they do seek care, if they do.

One Blue Ridge SAP program participant, “B,” while admitted inpatient for eight weeks and placed on the protocol following neck surgery told a nurse he’d only stay if a Blue Ridge SAP volunteer (at that time a hospital employee) could visit him. That then-volunteer (an SAP employee at the time of this data collection) got clearance to visit and began to sit with the participant “*for even 15 minutes at a time*” every day, bringing him candy or a soda. Previously B had left the same hospital against medical advice (AMA) repeatedly due to the protocol but the staff person said, “*just a little human contact and treatment was enough to keep him there through his treatment.*” B told us that being at the local hospital was “*worse than prison*,” and said the worst part was not having visitors. He recounted that hospital staff told him he was “*lucky [to be alive]*” then muttered, almost as an afterthought, that he “*[didn’t] feel lucky*” while he was kept on the protocol.

Another program participant, “R,” needed to go to the hospital for treatment of a renal abscess. She told us that when she was about to be admitted she asked if she would be put on the protocol. When hospital staff told her yes she said she needed time to make arrangements at home – to be sure her things wouldn’t be stolen, and to let her mother know what was happening so her kids would be taken care of. R told us she went and secured her belongings, got clean clothes, called her kids and mother so they would know where she was, then went back to the hospital when she felt prepared to be put on the protocol and cut off from all contact. After hearing R recount this experience, during an outreach shift observed during participant-observation for the grant evaluation, a Blue Ridge SAP staff person observed that:

*if R had a reasonable expectation of being treated like other patients, she could have made phone calls from the hospital to ask friends to make all those arrangements for her, rather than delaying treatment.*

Moreover, during participant-observation Blue Ridge SAP participants regularly came to outreach sites with badly infected wounds, abscesses, and stitches to be removed, seeking care from the nurse on staff because they did not want to go to or return to the local hospital for fear of being placed on the protocol. Local homeless services staff were clearly aware of the protocol as well: during participant-observation we observed case managers from a local housing case management program mention to Blue Ridge SAP staff that their clients will not go to the local hospital, saying it’s “*understandable*” given how PWUD were treated there.

SAP staff also described a lack of low-barrier access to primary care for PWUD: at the time of the grant evaluation most local primary care providers did not treat uninsured patients or did not have sliding-scale options, while primary care providers that did have uninsured or sliding-scale options had long wait times. This resulted in participants waiting a very long time to get medical care, or not getting it at all; and being much sicker or a medical condition being much more advanced by the time they received care. A Blue Ridge SAP staff person reported that every participant she referred to primary care at a local low-income clinic waited at least eight weeks for an intake appointment, observing, “*people in survival mode can’t think eight weeks ahead*.” SAP staff described that even participants established in local sliding-scale or subsidized primary care experienced long waits to be seen for acute issues and expressed concern that infections can become septic while waiting: one staff person talked about a participant, “M” who had symptoms of a urinary tract infection (UTI) but had to wait ten days for an appointment at a local low-income clinic where she was already an established patient. The SAP staff person stated that by the time M was seen her UTI had progressed to a kidney infection.

Meanwhile, some participants reported feeling pressured into starting medications for opioid use disorder (MOUD) at the one local low-income clinic that offered it when they did seek primary care, even if they did not meet criteria for opioid use disorder or were not primarily opioid users. Blue Ridge SAP staff described there otherwise being a widespread local emphasis on abstinence-only and recovery-oriented options that they believed contributed to PWUD internalizing abstinence-only/recovery messaging; which staff also felt could be stigmatizing and contribute to self-blame:

*we see a lot of self-blame [among participants] for being unhoused, for law enforcement harassment, for having HIV, for HCV… that self-blame is more common among people that have been in recovery, they have more internalized stigma…*

**Fig. 1:**
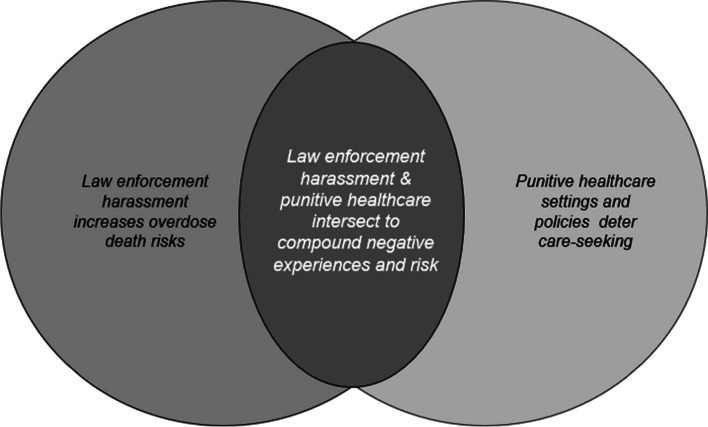
Schema: intersecting themes

Both thematic areas described above converged in a third overarching thematic area; elaborated in Fig. 1.

### Law enforcement harassment & punitive healthcare intersect to compound negative experiences and risk

Blue Ridge SAP participants and staff described intersections between law enforcement harassment and punitive healthcare policies and experiences that together compounded negative experiences for PWUD at risk for justice involvement and exacerbated avoidance of care-seeking and fear of law enforcement interactions. At times, the punitive nature of healthcare settings were nearly conflated with carceral settings, and punitive healthcare policies seen as extensions of law enforcement. Widespread awareness among SAP participants of the local hospital’s protocol for PWUD combined with their awareness of local law enforcement routinely responding to 911 overdose and medical emergency calls reinforced widespread underlying distrust of doctors and nurses based on past negative healthcare experiences that were already common among program participants. These concerns were further compounded by what participants and staff described as a local lack of distinction in behavior between law enforcement. and healthcare facilities, which further added to mistrust and avoidance of care among justice-involved PWUD. For example, fear of being “*put on the protocol*” combined with fear of hospital staff running patients for warrants (which participants perceived occurs despite being a violation of hospital policy) means many program participants would not go to the local hospital even when experiencing severe symptoms. SAP staff reported that participants were afraid of being arrested at the Emergency Department of the local hospital, based on participants’ perceptions that the local hospital runs PWUD for warrants. Participants described concerns that calling 911 for an overdose or medical emergency would result in being run for warrants and going to jail instead of being taken to the hospital - in many cases such concerns were based on personal experiences they described of precisely this occurring. SAP staff interviewed for the grant evaluation attributed such concerns, in part, to program participants being unaware of the specifics of “dual dispatch,” the policy in effect at the local hospital in which local law enforcement can issue a warrant to the hospital if they become aware a person with an outstanding warrant is at the hospital, for example if the person was transported by Emergency Services with police present. Blue Ridge SAP staff explained in grant evaluation interviews that in the case of dual dispatch the hospital may opt to detain a patient for arrest if law enforcement issue the warrant while they are still at the hospital. SAP staff recounted multiple times this had happened to program participants, speculating that, to the person being arrested, it would not be evident that the hospital had detained them on a warrant issued by law enforcement and binding on the hospital versus the warrant being issued or run by hospital staff directly. As a Blue Ridge SAP staff person said, “*when justice-involved PWUD can’t differentiate between medical care and law enforcement, it’s a problem*.”

In another example of avoidance of care-seeking due to fear of and conflation of law enforcement with the medical system, during participant-observation for the grant evaluation a program participant came to an outreach site with visible limb swelling and open sores; the SAP staff nurse suspected a systemic infection. The participant was reluctant to go to hospital for treatment stating she believed hospital staff would “*run a warrant check*.” She told us when she had recently been at the hospital to deliver a baby, she was arrested soon after. Her perception was hospital staff might have run a warrant check at the time, or that a mandatory Department of Social Services notification resulting from the participant being identified as having used non-prescribed substances during pregnancy (Leiner et al., 2021) might have resulted in the arrest. Either way, the participant associated receiving care at that hospital with her subsequent arrest. During an informal interview for the grant evaluation the same Blue Ridge SAP participant also told us she had left the hospital against medical advice (AMA) a previous time she was admitted for a cardiac infection because she was placed on the protocol.

### Surveillance increases health risks

In informal interviews for the grant evaluation Blue Ridge SAP staff reported and we observed during participant-observation that seemingly disproportionate law enforcement surveillance in public housing areas, and of unsheltered program participants, appeared to unevenly increase health risks for program participants that described justice-involvement in the form of frequent paraphernalia arrests; tickets, arrests, and short-term jail stays for panhandling and loitering; and arrests at the scene of overdoses. SAP staff and program participants alike described the presence of law enforcement, especially around public housing and in areas where unsheltered PWUD gathered, as a deterrent to people at high risk for arrest obtaining overdose and infection prevention supplies from Blue Ridge SAP, and/or being especially concerned that doing so would jeopardize their legal and housing/shelter circumstances. Blue Ridge SAP staff described, in informal interviews and focus groups for the grant evaluation, how these dynamics particularly affected BIPOC participants, precariously housed or unsheltered participants, and those at the intersection of more than one of these markers of structural marginalization. SAP staff recounted the increased presence of law enforcement near Blue Ridge SAP outreach sites, particularly outreach sites recently established in public housing areas – which are disproportionately populated by BIPOC residents as compared to the surrounding county population. SAP staff reported and we observed during participant-observation that, particularly as SAP outreach in public housing areas was just beginning under the grant funding, and with significant law enforcement presence, participants in public housing were initially hesitant about coming to the outreach unit for overdose and infection prevention supplies. At that time, SAP staff reported, and we observed during participant-observation, that BIPOC participants appeared to feel a greater need to be discreet and seemed more reluctant to give demographic information such as dates of birth and initials (which are confidentially recorded for the SAP’s internal supply distribution and participant characteristic tracking and monitoring, requested and confidentially stored to maintain records of unique participants for reporting de-identified distribution statistics as required by the state and funders). SAP staff also reported, and we observed during participant-observation, that many BIPOC participants asked outreach staff and volunteers more questions about risks for paraphernalia charges and limited immunity clauses in the syringe exchange and Good Samaritan laws; tended to be more likely to ask for an additional plastic bag to cover the standard paper bag that SAP supplies come in; and seemed more intent on keeping any safer drug consumption supplies not covered by limited immunity separate from those that are even when walking just short distances back to their public housing apartments nearby.

Early on in establishing mobile outreach at public housing areas, SAP staff reported they noticed less sustained interaction and shorter conversations with the most structurally marginalized program participants, especially BIPOC residents of public housing. One Blue Ridge SAP staff person said, in an informal interview for the grant evaluation, “*in [public housing] we have longer conversations with white participants, and shorter interactions with Black participants. and when Black participants are arrested [in violation of limited immunity] they are held in jail much longer*.”

Blue Ridge SAP staff reported that BIPOC participants sometimes requested SAP staff collect used syringes at their apartment door nearby (rather than bringing used syringes to the outreach unit for disposal, as is more standard) expressing reluctance to carry the used supplies in public for fear of a paraphernalia charge (although limited immunity should cover return of used supplies).We also observed that more BIPOC participants at public housing-based outreach sites requested the intranasal form of naloxone rather than the intramuscular (IM) kits, that come with syringes. During participant-observation, when a noticeable trend emerged that BIPOC participants were hesitant to carry IM kits that contain syringes, we engaged SAP staff and volunteers in discussion about why this could be and they concluded it could potentially be due in part to concerns about local law enforcement violating the limited immunity clause of the syringe exchange law – based on program participant reports of people being arrested for paraphernalia due to unused naloxone syringes in overdose prevention kits. (Alternatively, BIPOC program participants at public housing sites were more likely to engage in non-injection drug use and report more discomfort with syringes, overall, thus potentially being simply more comfortable with intranasal naloxone).

As described previously, Blue Ridge SAP staff also perceived that the repeated ticketing of especially unsheltered participants, leading to warrants, arrests, and short periods in jail (when people could not afford bail) each increased these program participants’ overdose risks. One SAP staff person described “*overpolicing*” in public housing and in areas where unsheltered participants gathered as, “*constant surveillance but not help… it’s easier to count when there aren’t cops at outreach than when they are*.” Another staff person said, “c*ops being near outreach sites is similar to the early days of harm reduction, cops posted up reduces access to services, it causes participants to look over their shoulder*.”

In another example of this, a white unsheltered participant previously referred to as B told Blue Ridge SAP staff he had never been arrested prior to becoming homeless but had recently gotten 12 panhandling tickets in one week in the area near an outreach site. He told us this rapid accumulation of tickets resulted in a warrant leading to arrest and time in jail. B described the following interaction when receiving one of those tickets, to an SAP staff person:

“*Why are you begging for money?*” – law enforcement officer.

“*Would you rather I steal?*” – B.

“*There’s free food at [local soup kitchen]*.” – law enforcement officer.

“*Is food all YOU need to live?*” – B.

SAP staff expressed concern that the days B spent in jail on the warrant resulting from accumulated panhandling tickets increased his risk for a fatal overdose, which was already high given large amounts of fentanyl then circulating in the local drug supply.

## Discussion

In this discussion, we focus primarily on SAP participant and staff perspectives about the broader context in which grant activities occurred, rather than on grant deliverables. In the year preceding and into the first year of the COVID-19 pandemic, PWUD and staff at the community-based SAP where they obtained services each reported a perception that local law enforcement harassment increased overdose death and infectious disease risks and that punitive local healthcare settings and policies acted as deterrents to PWUD seeking healthcare. Together our analysis of these distinct perspectives informed an overarching thematic finding that law enforcement harassment and punitive healthcare settings and policies intersected to compound negative law enforcement and healthcare experiences, thereby increasing overdose and health risks for local PWUD already at increased risk of justice involvement. In sum, we argue that an inherently justice-involved population, PWUD, often experienced local healthcare as carceral and that experiences with local law enforcement and carceral-like healthcare experiences interfered with what could otherwise have been the full benefits of community-based public health resources in the form of syringe services and infectious disease prevention supply distribution.

Moreover, as evident in SAP participants’ reports of constant surveillance and with the frequency and constant specter of law enforcement harassment for PWUD served by Blue Ridge SAP, nearly all SAP participants could be considered justice-involved. Most have been arrested for paraphernalia at some time, if not frequently - in violation of the limited immunity statute of NC’s syringe exchange law. Others have been incarcerated previously, and/or have outstanding warrants, and, confirming the body of existing research on the topic (e.g., Beletsky et al., 2015, etc.) thus are even more leery of law enforcement interactions. All participants are likely aware of the probability that fellow PWUD would prefer to avoid interacting with law enforcement, and again confirming earlier studies, are fearful to seek emergency assistance on behalf of a fellow participant in the context of an overdose or medical emergency.

Our finding that local law enforcement harassment likely increases overdose death and infectious disease risks for highly justice-involved PWUD in this Southern Appalachian settings is consistent with existing national and regional literature (Baker et al., 2020; Beletsky et al., 2015; Carpenter et al., 2022; Davis et al., 2019)**.** With steadily increasing rates of overdose deaths in Appalachia and nationwide (CDC, 2021), accompanied by substantial federal policy and funding investment in reducing overdose and supporting harm reduction (Biden, 2021), any law enforcement behavior that undermines the success of harm reduction programs is not only counter-productive to public health but has the potential to be deadly. Municipal, county, and state law enforcement agencies, and their individual patrol officers, must be trained and held accountable to follow relevant syringe exchange limited immunity and Good Samaritan laws. In many cases, existing state-level limited immunity from prosecution laws cover only injecting equipment, not other safer drug consumption supplies such as snorting or smoking equipment, both of which have greater potential to reduce overdose deaths and are also important for reducing infectious disease spread. Such non-injection supplies are also more widely used by non-white SAP participants, who are more often targeted by law enforcement. As part of the grant evaluation Blue Ridge SAP staff reported that beginning to offer safer smoking supplies, and beginning distribution in a public housing area with a large BIPOC population, increased the number of BIPOC program participants significantly over what it had previously been – and that these program participants reported greater levels of and fears about law enforcement harassment that had otherwise deterred them from attending the SAP. Limited immunity thus must also be expanded beyond just injecting supplies to truly reduce the threat of law enforcement to the health and wellbeing of PWUD and maximize the full benefits of harm reduction while also improving racial equity in overdose death and infectious disease prevention.

Likewise, our finding that punitive local healthcare settings and policies acted as deterrents to care-seeking among PWUD in this Southern Appalachian setting is also consistent with existing national and regional literature on the negative health effects of stigmatizing and discriminatory healthcare experiences and policies affecting PWUD (Jafari et al., 2015; McNeil et al., 2014; Meyerson et al., 2021; Paquette et al., 2018). We found participants’ previous experiences with some local healthcare providers’ approaches to encouraging SUD treatment even when not applicable to a participant’s needs could become a deterrent to later seeking SUD treatment when they did want it. Moreover, such coercion around treatment also became a deterrent to seeking other healthcare that *was* wanted or needed such as HCV treatment, primary care, or prenatal care. It is important to note that in the community where Blue Ridge SAP provided harm reduction services, and healthcare referrals, the most accessible providers of low-income, sliding-scale, or no-cost healthcare and SUD treatment were often co-located - thus SAP participants were unlikely to differentiate between a coercive or discriminatory SUD treatment provider, and providers of other forms of healthcare in the same physical clinic. This is an important consideration for healthcare providers treating low-income populations that include PWUD, and justice-involved populations, to keep in mind.

The extent to which our participants’ descriptions of law enforcement harassment and punitive healthcare settings and policies overlapped and intersected to compound the effects of each, further increasing their overdose and other health risks, speaks to the need for expansion of limited immunity laws related to supplies obtained from SAPs, and for a clearer separation between healthcare and carceral systems. For harm reductionists working in direct service, the implications of these findings are likely unsurprising: syringe service staff working on the ground are well aware that program participants benefit from education about limited immunity laws, including education about the limitations of such laws; and are often reluctant to seek medical and emergency care. Identifying sources of medical care that are explicitly disconnected from carceral systems to which participants can be referred, and providing as much care onsite as possible (e.g., wound care, partnering with mobile primary care clinics, etc.), may be key. Offering ample naloxone and frequent overdose reversal training is especially critical for all organizations serving justice-involved populations, given the likelihood that these clients may avoid seeking emergency assistance for fear of law enforcement interaction. For law enforcement and healthcare audiences, a key takeaway should include the necessity of disentangling the systems in which they work to reduce the harms of policing for justice-involved people at increased risk of overdose and in need of healthcare. Even if unintended, police surveillance and allowing law enforcement presence and carceral policies within healthcare settings each pose threats to the health and survival of PWUD already at greater risk for justice involvement by virtue of the criminalization of substance use in the U.S.

## Limitations

The dynamics of COVID-19 that directly overlapped with much of the grant period and the ways the pandemic measurably increased overdose risks and infectious disease vulnerability and spread inevitably affected the grant evaluation, and thus, the data that informed these larger findings. Another limitation is that the secondary program data provided by Blue Ridge SAP was likely incomplete, and may not have reflected demographic (race/ethnicity) data for all unique participants seen during the funded period – only those data for which all variables were included were analyzed. Other limitations include that the data analyzed and presented here were initially collected for evaluation purposes, and thus primarily focused on grant deliverables. However, as described above, because a modified Grounded Theory approach was used it was possible for larger thematic findings to emerge and inform larger research questions. To maximize validity of the grant evaluation, an outside evaluator conducted the majority of data collection and analysis, but triangulated the findings with program staff.

## Conclusion & recommendations

Despite the COVID-19 pandemic beginning nearly at the same time as the funded grant period, Blue Ridge SAP conducted most of the proposed funded activities or was able to pivot and develop alternate activities to address similar needs for a population of highly marginalized PWUD at risk for justice involvement. An evaluation of the grant that funded these activities revealed program participant concerns about and lived experiences with the larger structural and policy environment of the settings where the evaluation data were collected and the surrounding region. Thematic findings from program staff and program participant evaluation data suggest that overlap between overpolicing of SAP participants, especially BIPOC public housing residents, and unsheltered PWUD, and punitive healthcare policies, continue to systematically disadvantage PWUD and increase risks for overdose, infectious disease, worsened health outcomes, and human and civil rights violations. Continued investment in and support for Blue Ridge SAP and programs like it are needed to maintain and maximize the proven benefits of harm reduction. However, broader community, regional, and national advocacy to reduce structural and systemic sources of suffering and oppression affecting marginalized PWUD at great risk for justice-involvement, overdose, infectious disease, and other negative health and social consequences of criminalized substance use, are greatly needed to address the larger upstream structural and policy causes. Particular attention must be paid to the compounding negative health effects of law enforcement harassment and punitive healthcare policies and settings.

## Data Availability

Under the terms of our human subjects protocol and given the confidential and sensitive nature of the topics, the data and materials are not publicly available.

## Abbreviations

AMA: against medical advice
BIPOC: Black, Indigenous, and People of Color
HCV: Hepatitis C
HIV: Human Immunodeficiency Virus
IM: intramuscular
NC: North Carolina
PWUD: people who use drugs
SAP: Syringe Access Program, also known as Syringe Services Program
US: United States

## References

[CR1] Ahlner J, Holmgren A, Jones AW (2016). Demographics and post-mortem toxicology findings in deaths among people arrested multiple times for use of illicit drugs and/or impaired driving. Forensic Science International.

[CR2] Baker P, Beletsky L, Avalos L, Venegas C, Rivera C, Strathdee SA, Cepeda J (2020). Policing practices and risk of HIV infection among people who inject drugs. Epidemiologic Reviews.

[CR3] Banta-Green CJ, Beletsky L, Schoeppe JA, Coffin PO, Kuszler PC (2013). Police officers’ and paramedics’ experiences with overdose and their knowledge and opinions of Washington State’s drug overdose–naloxone–good Samaritan law. Journal of Urban Health.

[CR4] Barnes JC, Jorgensen C, Beaver KM, Boutwell BB, Wright JP (2015). Arrest prevalence in a National Sample of adults: The role of sex and race/ethnicity. American Journal of Criminal Justice.

[CR5] Beletsky L, Cochrane J, Sawyer AL, Serio-Chapman C, Smelyanskaya M, Han J, Robinowitz N, Sherman SG (2015). Police encounters among needle exchange clients in Baltimore: Drug law enforcement as a structural determinant of health. American Journal of Public Health.

[CR6] Biden, J. (2021). Statement by president Joe Biden on surpassing 100,000 American overdose deaths in the past year. *White House Briefing Room* https://www.whitehouse.gov/briefing-room/statements-releases/2021/11/17/statement-by-president-joe-biden-on-surpassing-100000-american-overdose-deaths-in-the-past-year/.

[CR7] Binswanger IA, Glanz JM (2018). Potential risk window for opioid overdose related to treatment with extended-release injectable naltrexone. Drug Safety.

[CR8] Binswanger IA, Nguyen AP, Morenoff JD, Xu S, Harding DJ (2020). The association of criminal justice supervision setting with overdose mortality: A longitudinal cohort study. Addiction.

[CR9] Bohnert A, Nandi A, Tracey M, Cerda M, Tardiff K, Vlahov D, Galea S (2011). Policing and risk of overdose mortality in urban neighborhoods. Drug and Alcohol Dependence.

[CR10] Brinkley-Rubinstein L, Cloud D, Drucker E, Zaller N (2018). Opioid use among those who have criminal justice experience: Harm reduction strategies to lessen HIV risk. Current HIV/AIDS Reports.

[CR11] Brinkley-Rubinstein L, Zaller N, Martino S, Cloud DH, McCauley E, Heise A, Seal D (2018). Criminal justice continuum for opioid users at risk of overdose. Addictive Behaviors.

[CR12] Bryce, A., Brown, H., Goldstein, R., & Ostrach, B. (2021). Druge Use Harm Reduction Best Practices for Western North Carolina: Contextually Informed, Locally Specific Evidence-Based Recommendations for Funders & Practitioners.

[CR13] Carpenter, D. M., Zule, W. A., Hennessy, C. M., Evon, D. M., Hurt, C. B., & Ostrach, B. (2022). Factors Associated with Perceived Ease of Access to Syringes in Appalachian North Carolina. *The Journal of Rural Health*. 10.1111/jrh.12698 PMC9772148 35819251

[CR14] Carroll, J., Green, T., & Noonan, R. (2018). *Evidence-based strategies for preventing opioid overdose: What’s working in the United States, 2018* (p. 40). Centers for disease control. https://www.cdc.gov/drugoverdose/pdf/pubs/2018-evidence-based-strategies.pdf

[CR15] Carroll JJ, Ostrach B, Wilson L, Dunlap JL, Getty R, Bennett J (2021). Drug induced homicide laws may worsen opioid related harms: An example from rural North Carolina. International Journal of Drug Policy.

[CR16] Caulkins JP, Reuter P (2021). Ending the war on drugs need not, and should not, involve legalizing supply by a for-profit industry. The American Journal of Bioethics.

[CR17] CDC (2020). Recent HIV clusters and outbreaks across the United States among people who inject drugs and considerations during the COVID-19 pandemic.

[CR18] CDC (2021). *Drug Overdose Deaths in the U.S. Top 100,000 Annually*.

[CR19] Charmaz K (2006). Constructing grounded theory: A practical guide through qualitative research.

[CR20] Creswell JW (2013). Qualitative inquiry and research design: Choosing among five approaches.

[CR21] Davis SM, Kristjansson AL, Davidov D, Zullig K, Baus A, Fisher M (2019). Barriers to using new needles encountered by rural Appalachian people who inject drugs: Implications for needle exchange. Harm Reduction Journal.

[CR22] Des Jarlais DC, Perlis T, Arasteh K, Torian LV, Hagan H, Beatrice S, Smith L, Wethers J, Milliken J, Mildvan D, Yancovitz S, Friedman SR (2005). Reductions in hepatitis C virus and HIV infections among injecting drug users in new York City, 1990–2001. AIDS.

[CR23] Dixon P (2023). A refresher on North Carolina’s needle exchange law and other harm reduction immunities.

[CR24] G.S.90-113.22. Possession of drug paraphernalia, (2019). https://www.ncleg.gov/EnactedLegislation/Statutes/PDF/BySection/Chapter_90/GS_90-113.22.pdf

[CR25] Hamilton LK, Wheeler-Martin K, Davis CS, Martins SS, Samples H, Cerdá M (2022). A modified Delphi process to identify experts’ perceptions of the most beneficial and harmful laws to reduce opioid-related harm. International Journal of Drug Policy.

[CR26] Jafari S, Joe R, Elliot D, Nagji A, Hayden S, Marsh DC (2015). A Community Care Model of Intravenous Antibiotic Therapy for Injection Drug Users with Deep Tissue Infection for “Reduce Leaving Against Medical Advice”. International Journal of Mental Health and Addiction.

[CR27] Kaiser Family Foundation. (2017). Rurality in the United States, by county (using index of relative rurality). https://public.tableau.com/views/RuralityintheU_S_byCounty/RuralityintheUnitedStatesbyCounty?%3Aembed=y&%3AshowVizHome=no&%3Adisplay_count=y&%3Adisplay_static_image=y&%3AbootstrapWhenNotified=true&%3Alanguage=en&:embed=y&:showVizHome=n&:apiID=host0#navType=0&navSrc=Parse

[CR28] Kattakuzhy S, Rosenthal E (2020). To eliminate hepatitis C in people who inject drugs.

[CR29] Leiner C, Cody T, Mullins N, Ramage M, Ostrach BMM (2021). “The elephant in the room;” a qualitative study of perinatal fears in opioid use disorder treatment in southern Appalachia. BMC Pregnancy and Childbirth.

[CR30] Linas BP, Savinkina A, Madushani R, Wang J, Yazdi GE, Chatterjee A, Walley AY, Morgan JR, Epstein RL, Assoumou SA (2021). Projected estimates of opioid mortality after community-level interventions. JAMA Network Open.

[CR31] McNeil R, Small W, Wood E, Kerr T (2014). Hospitals as a ‘risk environment’: An ethno-epidemiological study of voluntary and involuntary discharge from hospital against medical advice among people who inject drugs. Social Science & Medicine.

[CR32] Meyerson, B. E., Russell, D. M., Kichler, M., Atkin, T., Fox, G., & Coles, H. B. (2021). I don’t even want to go to the doctor when I get sick now: Healthcare experiences and discrimination reported by people who use drugs, Arizona 2019. *International Journal of Drug Policy*, 103112. 10.1016/j.drugpo.2021.103112. 10.1016/j.drugpo.2021.103112 33461838

[CR33] Naumann RB, Durrance CP, Ranapurwala SI, Austin AE, Proescholdbell S, Childs R, Marshall SW, Kansagra S, Shanahan ME (2019). Impact of a community-based naloxone distribution program on opioid overdose death rates. Drug and Alcohol Dependence.

[CR34] Ostrach B, Buer L-M, Armbruster S, Brown H, Yochym G, Zaller N (2021). COVID-19 and rural harm reduction challenges in the US Southern Mountains. The Journal of Rural Health: Official Journal of the American Rural Health Association and the National Rural Health Care Association.

[CR35] Paquette CE, Syvertsen JL, Pollini RA (2018). Stigma at every turn: Health services experiences among people who inject drugs. International Journal of Drug Policy.

[CR36] Ranapurwala SI, Shanahan ME, Alexandridis AA, Proescholdbell SK, Naumann RB, Edwards D, Marshall SW (2018). Opioid overdose mortality among former North Carolina inmates: 2000–2015. American Journal of Public Health.

[CR37] Rouhani S, Schneider KE, Rao A, Urquhart GJ, Morris M, LaSalle L, Sherman SG (2021). Perceived vulnerability to overdose-related arrests among people who use drugs in Maryland. International Journal of Drug Policy.

[CR38] Singer M (2007). Drugging the poor: Legal and illegal drugs and social inequality.

[CR39] Singer M, Page JB (2016). The social value of drug addicts: Uses of the useless.

[CR40] Singer M, Ziegler J (2017). The role of drug user stigmatization in the making of drug-related Syndemics.

[CR41] Small D, Glickman A, Rigter G, Walter T (2010). The Washington needle depot: Fitting healthcare to injection drug users rather than injection drug users to healthcare: Moving from a syringe exchange to syringe distribution model. Harm Reduction Journal.

[CR42] Strike C, Miskovic M (2018). Scoping out the literature on mobile needle and syringe programs—Review of service delivery and client characteristics, operation, utilization, referrals, and impact. Harm Reduction Journal.

[CR43] USDA Economic Research Service (2020). *Documentation 2010 Rural-Urban Commuting Area (RUCA) Codes*.

[CR44] Wagner KD, Simon-Freeman R, Bluthenthal RN (2013). The association between law enforcement encounters and syringe sharing among IDUs on skid row: A mixed methods analysis. AIDS and Behavior.

[CR45] Wodak A, Cooney A, World Health Organization (2004). Effectiveness of sterile needle and syringe programming in reducing HIV/AIDS among injecting drug users.

