# Correction: “When people who use drugs can’t differentiate between medical care and cops, it’s a problem.” Compounding risks of law enforcement harassment & punitive healthcare policies

**DOI:** 10.1186/s40352-024-00270-z

**Published:** 2024-04-20

**Authors:** Bayla Ostrach, Vanessa Hixon, Ainsley Bryce

**Affiliations:** 1 Boston University School of Medicine, Fruit of Labor Action Research & Technical Assistance, LLC, Fairview, NC USA; 2 Appalachian Medical Solidarity, Asheville, NC USA; 3 Holler Harm Reduction, Marshall, NC USA

**Correction:** ***Health Justice*** **12, 3 (2024)**

10.1186/s40352-023-00256-3

Following publication of the original article (Ostrach et al., 2024), the authors clarified that, in order to preserve participant confidentiality, the specific name of the Institutional Review Board that determined the study protocol exempt from further review should have been suppressed in the final article.

The original article (Ostrach et al., 2024) has been updated.
